# Evidence-based health human resources planning and medical professionals’ education in Iran

**DOI:** 10.1186/1472-6963-14-S2-P123

**Published:** 2014-07-07

**Authors:** Shima Tabatabai, Seyed Amir Mohsen Ziaee, Nasser Simforoosh

**Affiliations:** 1 School of Medical Education, Shahid Beheshti University of Medical Sciences, Tehran, lran; 2 Shahid Beheshti University of Medical Sciences, Tehran, Iran; 3 Ministry of Health and Medical Education, Tehran, Iran

## Background

There is a global crisis constituting severe shortages of health professionals and mal-distribution of health human resources. There are some important mega trends that affect this challenge:

• Populations are aging and becoming more urbanized.

• Non-communicable diseases, nutrition-related, and maternity-related causes of death, mental health disorders and chronic diseases are growing.

• People today are better educated and more assertive and enjoy greater access to information. The shift is toward shared medical decision making.

• The health human resource workforce effort is reducing.

Each of these transitions is a powerful force for change in health workforce planning, the roles of health professionals, and the design of medical professional education. Every country will have to respond to these global pressures for changes.

The Deputy Ministry for Education of Iran Health and Medical Education Ministry is working to progress health workforce reform to address the challenges of providing a skilled, innovative and flexible health professional education and planning in Iran.

In 2013 the medical education Deputy, designed a national projections for general practitioners, medical specialists and sub-specialists over a planning horizon to 2025, to ensure Iran health Human Resources meets the community’s needs.

## Materials and methods

After a systematic review of existing workforce planning, forecasting, and foresight methodologies, we developed a new model for foresight of the Health Supply and Medical Professionals Education in Iran.

In the first phase, we conducted linear trend analyses by using the first-hand historical data of medical workforce in Iran through 1979-2012, then, we forecasted the trend out to next 10 years. In the 2^nd^ phase we used qualitative foresight methods. Panel expert and Scenario modeling are the main methods of our study to explore the implications of possible alternative futures.

## Results

With trend analysis we showed that there has been significant growth of the absolute numbers of the health workforce over the past 3 decades. The findings revealed that the Iranian health workforce is not sustainable over the next 10 years, with a need for long-term reforms by government, professions and the medical education and postgraduate training sector for an effective health workforce. The main policy levers identified to achieve change were innovation and reform, training capacity and efficiency and workforce distribution.

## Conclusion

This national project is an ongoing process and will continue to develop health workforce future studies incorporating data and methodology improvements to support Evidence-Based Health Human Resources planning and Medical Professionals Education changes In Iran.

